# Workplace Violence among British Columbia Nurses Across Different Roles and Contexts

**DOI:** 10.3390/healthcare8020098

**Published:** 2020-04-14

**Authors:** Farinaz Havaei, Maura MacPhee, Andy Ma

**Affiliations:** 1 School of Nursing, The University of British Columbia, Vancouver, BC V6T 2B5, Canada; Maura.MacPhee@ubc.ca; 2 School of Educational and Counselling Psychology and Special Education, The University of British Columbia, Vancouver, BC V6T 1Z4, Canada; andytfma@mail.ubc.ca

**Keywords:** workplace violence, types, sources, roles, sector, geographical region, specialty, nursing, patients, family, visitors

## Abstract

Workplace violence in healthcare settings is on the rise, particularly against nurses. Most healthcare violence research is in acute care settings. The purpose of this paper is to present descriptive findings on the prevalence of types and sources of workplace violence among nurses in different roles (i.e., direct care, leader, educator), specialties, care sectors (i.e., acute, community, long-term care) and geographic contexts (i.e., urban, suburban, rural) within the province of British Columbia (BC), Canada. This is a province-wide survey study using a cross-sectional descriptive, correlational design. An electronic survey was emailed by the provincial union to members across the province in Fall 2019. A total of 4462 responses were analyzed using descriptive and chi-square statistics. The most common types of workplace violence were emotional abuse, threats of assault and physical assault for all nursing roles and contexts. Findings were similar to previous BC research from two decades ago except for two to ten times higher proportions of all types of violence, including verbal and physical sexual assault. Patients were the most common source of violence towards nurses. Nurses should be involved in developing workplace violence interventions that are tailored to work environment contexts and populations.

## 1. Introduction

Compared to employees in other industries, healthcare workers have a four-fold higher rate of exposure to workplace violence [1]. As the largest healthcare workforce and an overwhelmingly female dominant profession, nurses are more prone to workplace violence, particularly in constrained healthcare environments where workload management and staffing inadequacies are daily challenges [1,2]. Although the long-term consequences of workplace violence for providers, patients, the healthcare organization and the society at large have been relatively well studied [3], there is a gap in our understanding of different typologies and sources of workplace violence towards nurses across a variety of roles, specialties, sectors and geographical areas. To date, the majority of workplace violence research has been conducted with direct care nurses from urban acute care settings [4,5,6,7], with limited research on the state of workplace violence among non-direct care providers, such as nursing leaders and educators, and nurses in long-term care or community care sectors and/or healthcare settings across geographical areas (e.g., urban, rural). For example, violence rates in acute care settings have also been attributed to the close, frequent contact direct care nurses have with patients and families [2,4,6]. Less is known about violence rates in other healthcare sectors with nurses in roles that require less frequent, direct contact with patients and families. Moreover, previous healthcare violence research used simplistic operational definitions of workplace violence [5,8,9] or was conducted with data collected over two decades ago [4,6,9]. Because the consequences of workplace violence vary for victims depending on its type and source [10], describing workplace violence with respect to the multiple factors associated with nursing care delivery is an urgent undertaking. The purpose of this study was to examine the prevalence of workplace violence, in terms of typologies and sources, towards nurses in different roles and care delivery contexts in British Columbia (BC), Canada. 

Workplace violence encompasses the full range of acts and threats of physical violence against employees, from threatening or intimidating behaviors, harassment and verbal abuse to physical assaults at the worksite [11]. Workplace violence is a complex phenomenon with nuanced distinctions that have been difficult to isolate and measure. The World Health Organization, for instance, classifies workplace violence into two categories of physical and emotional violence [12]. Physical violence is described as the use of physical force that causes physical and psychological harm and emotional violence refers to the use of non-physical means that result in psychological harm [12]. These broad classifications fail to effectively capture the range of types of violence, particularly non-physical types of violence, such as emotional abuse. Hence, more research is needed on the prevalence of specific types of workplace violence among nurses. 

One of the most recent comprehensive reviews of nurse exposure to workplace violence examined 136 international studies with prevalence rates for physical and non-physical violence, bullying, and sexual harassment in an aggregated sample of 151,347 nurses [7]. This review found that 32% of nurses worldwide were exposed to physical violence, 63% to non-physical violence, 48% to bullying, and 18% to sexual harassment. Nurses had the highest violence exposure rates in emergency departments (physical = 50%, non-physical = 81), mental health (physical = 55%, non-physical = 73%) and long-term care settings (physical = 46%, non-physical = 34%). The highest prevalence of sexual harassment was in psychiatric settings (30%), followed by nurses working in hospitals (19%). Despite important knowledge gleaned from this quantitative review, the study failed to differentiate between nursing roles (e.g., direct and non-direct care); nurses were classified by world regions (i.e., Anglo, Asia, Europe, Middle East); and prevalence rates were based on considerable variation in how violence was operationalized and measured across studies. The most common types of violence reported in the review papers were physical violence, non-physical violence, bullying and sexual harassment. The review, therefore, ‘fit’ data into these four violence categories, acknowledging differences in how violence was defined and assessed. 

In this study’s country context, Canada, workplace violence seems to be particularly prevalent in BC. A 2005 national survey on the work and health of nurses found that BC nurses reported exposure above national averages for physical (33% vs. 29%) and emotional violence (50% vs. 44%). This research only used two categories of violence [9]. Two earlier Canadian studies used five specific categories of violence, but these studies were conducted with data collected over two decades ago [4,6]. 

In addition to the factors mentioned above that influence prevalence rates of workplace violence against nurses, violence research needs to include the source of violence and work environment factors associated with violence. Workplace violence is classified based the source or the instigator of violence. Instigators are categorized as: clients of the victim (e.g., patients, families), other employees working in the same organization as the victim (e.g., nursing colleagues, management), individuals who have personal relations with the victim (e.g., spouse), and individuals with no relation to the victims or the organization (e.g., stranger) [13]. According to previous research, the majority of workplace violence in healthcare is attributed to patients and their families/visitors. For example, the aforementioned international review reported that a majority of physical and non-physical workplace violence was respectively instigated by patients (64% and 54%) and families/visitors (30% and 47%) followed by organizational staff such as nursing colleagues, physicians and supervisors (2%–6% and 22%–39%) [7]. 

Workplace characteristics, such as lack of resources, inadequate staffing, heavy workload and long wait times are often the root causes of patient aggression towards healthcare providers [5,14,15,16]. A recent study, for example, found associations between nurses’ heavy workloads, patient/family complaints and violence escalation [5]. Other research has established differences in adequacy of nurse staffing and workload management between healthcare sectors (e.g., acute care, long-term care) [17] and geographical regions (e.g., urban, rural) [18,19,20]. Violence research with nurses, therefore, needs to consider the variety of contexts that may influence acts of violence against them. 

To summarize, the majority of previous workplace violence research was conducted with direct care nurses from urban acute care settings. There is limited evidence on the prevalence of workplace violence among nurses in non-direct care roles, working outsides of hospitals in different healthcare sectors and urban/rural regions. This study addressed previous research limitations and included other workplace factors related to violence, specifically the source or instigator of violence and work environment characteristics. Therefore, the purpose of this study was to establish a comprehensive baseline of workplace violence in a Canadian province with some of the highest reported violence rates against nurses. Baseline descriptive data will serve as a foundation for further exploration of the causal relationships most predictive of different types of violence against nurses based on their roles, specialties, healthcare sectors and urban/rural contexts.

## 2. Materials and Methods 

This was a province-wide study of BC nurses using a cross-sectional descriptive and correlational design. A weblink to an electronic survey was emailed by the provincial union to about members across the province inviting them to participate in a study of psychological health and safety in the workplace. To increase response rates, the survey was open for two months and email reminders were sent through the union e-news every week. The study was also advertised through the union’s social media and print advertisements. Additionally, respondents were included in a raffle draw for two Apple Watches. Respondents were informed that participation was completely voluntary, and survey completion indicated informed consent.

Overall, 5512 surveys were returned, reflecting an estimated response rate of 12%. Nurses were included in this study if they were actively working and fully completed the workplace violence questions. The final sample size was 4462. Ethics approval was obtained from the University Behavioral Research Ethics Board (approval number: H18-02724).

### 2.1. Measures 

*Workplace violence typologies* were measured by a single question that asked about the frequency of exposure to five different types of workplace violence over the last year. This question was previously content validated and used in research with BC nurses [6]. The types of workplace violence were: (i) physical assault (e.g., being spit on, bitten, hit, pushed), (ii) threat of assault (e.g., verbal or written threats intending harm, aggressive behavior), (iii) emotional abuse (e.g., insults, gestures, humiliation, coercion), (iv) verbal sexual harassment (e.g., unwanted intimate questions or remarks of a sexual nature) and (iv) sexual assault (e.g., any forced physical sexual contact including forcible touching and fondling and forced sexual acts including forcible intercourse). The response options were rated on a seven-point scale ranging from never (0) to everyday (6). Response options were recoded to never (0) versus ever (1). 

*Workplace violence sources* were measured by asking a check-all-apply question from respondents who reported exposure to workplace violence. Respondents were asked to identify the source from a list of seven response options for each type of workplace violence they had experienced: (i) patients, (ii) family/visitors, (iii) physicians, (iv) nursing co-workers, (v) allied health, (vi) management, and (vii) other. This question was coded and analyzed in two ways: (a) to differentiate between respondents that identified a single source versus multiple sources and (b) to explore nurses’ responses in each source. For the former, nurses that identified more than one source for each type of workplace violence were recoded into a multiple source category. For the latter, nurses’ responses were maintained in their original form. This question was previously content validated and used in research with BC nurses [6].

*Workplace characteristics* were measured by a series of questions that asked nurses to identify their primary workplace in terms of healthcare sector (acute care, community care, long-term care) and geographical region (urban, suburban and rural). Acute care nurses were asked to identify the primary nursing specialty of their workplace using a check-all-apply question with 17 response options. As per previous nurse workplace violence research, response options were recoded into medical/surgical, critical care, emergency, psychiatry, and other specialties [6]. Nurses that identified more than one specialty were recoded into a multi-specialty category. These questions were content validated in previous research with BC nurses [21].

*Demographic characteristics* were measured using a series of questions that asked nurses to identify their age, gender (female, male, prefer not to describe), professional designation (licensed practical nurse, registered nurses, registered psychiatric nurses and dually registered nurses), education (diploma/certificate, undergraduate degree, graduate degree, other), years of nursing experience and role (direct care provider, nurse leader, nurse educator). 

### 2.2. Data Analysis

Data were analyzed using descriptive statistics such as frequencies, percentages and cross tabs. Chi square analysis was used to examine the difference in workplace violence across nursing roles, healthcare sectors, geographical areas and nursing specialties. Chi square analysis was not conducted for variables that resulted in cross tabs with empty cells, as empty cells violate an important assumption of this test statistic. The Statistical Package for Social Sciences for Windows 25.0 (SPSS Inc., Chicago, IL, USA) was used for data analysis.

## 3. Results

Table 1 shows respondents’ demographic and workplace characteristics. An overwhelming majority of the respondents were female (91%) registered nurses (RNs) (78%) with an age range of 25–54 years old (80%). About 50% of the respondents had an undergraduate degree with less than 11 years of nursing experience. Most nurses held a direct care role (90%) in urban (63%) acute care (74%) settings. A majority of acute care nurses worked in medical/surgical (35%), other (22%) and critical care specialties (18%).

Table 2 shows the prevalence of the types of workplace violence among BC nurses and across different nursing roles, healthcare sectors and geographical areas. The most common types of workplace violence were respectively emotional abuse (83%), threat of assault (78%), physical assault (67%), verbal sexual harassment (55%) and sexual assault (11%) among BC nurses. On average, BC nurses reported exposure to emotional abuse (*M* = 2.0, *SD* = 1.7) and threat of assault (*M* = 1.9, *SD* = 1.7) once a month, physical assault (*M* = 1.3, *SD* = 1.4) and verbal sexual harassment (*M* = 1.0, *SD* = 1.3) a few times a year; and sexual assault (*M* = *0*.2, *SD* = 0.5) rarely. 

While the most common types of workplace were respectively emotional abuse, threat of assault and physical assault, the least common types of workplace violence were sexual assault and verbal sexual harassment across all three nursing roles, healthcare sectors and geographical areas. With respect to nursing role, all types of workplace violence were more predominantly reported by direct care nurses (Range = 12%–84%) and nurse leaders (Range = 10%–79%) compared to educators (Range = 0%–60%) (χ^2^ (*24,462*) = 50.3–89.8, *p* < *0*.001). With respect to healthcare sector, all types of workplace violence were more predominantly reported by acute care (Range = 13%–85%) and long-term care nurses (Range = 16%–84%) compared to community care nurses (Range = 4%–72%) (χ^2^ (*24,462*) = 57.1–591.1, *p* < *0*.001). With respect to geographical area, there were no statistically significant differences in four types of workplace violence across urban, suburban and rural areas (χ^2^ (*24,443*) = 0.6–5.7, *p* > 0.05). An exception includes verbal sexual harassment that was most commonly reported by nurses in rural areas (61%) compared to their counterparts in urban (52%) and suburban (57%) areas (χ^2^ (*24,443*) = 22.5, *p* < *0*.001).

Table 3 shows the prevalence of the types of workplace violence among acute care nurses by nursing specialty. There were statistically significant differences in the prevalence of all types of workplace violence across acute care nursing specialties (χ^2^ (*53,260*) = 140.5–424.1, *p* < 0.001). Overall, workplace violence was most commonly reported by acute care nurses that worked in emergency departments (Range = 17%–97%), psychiatric (Range = 8%–96%) and medical/surgical (Range = 20%–91%) specialties. Nurses in critical care and other specialties were less commonly exposed to all types of workplace violence.

Table 4 shows the prevalence of the types of workplace violence by its sources (single vs. multiple) across nursing roles, healthcare sectors and geographical areas. A similar pattern was noted across all nursing roles and most healthcare sectors and geographical areas. While physical assault (Range: 70%–84%), verbal sexual harassment (Range: 64%–74%), and sexual assault (Range: 66%–78%), were most commonly initiated by patients, multiple perpetuators were most commonly reported for emotional abuse (Range: 60%–77%), and threats of assault (Range: 33%–53%). Exceptions included threats of assault in community and long-term care settings as well as rural areas where patients alone were identified as the most common single instigators of these types of violence. 

Table 5 similarly shows the prevalence of the types of workplace violence by sources across nursing roles, sectors and geographical areas, but without the grouping of responses that reported multiple sources of a workplace violence type. Therefore, the percentages in Table 5 report the prevalence of workplace violence for each source individually. Overall, patients were overwhelmingly the most common source of workplace violence for physical assault (Range: 94%–99%), verbal sexual harassment (Range: 76%–91%), and sexual assault (Range: 80%–89%) across roles, sectors, and geographical areas. For threats of assault, most commonly reported sources were patients (Range: 78%–96%) followed by family/visitors (Range: 33%–57%). Similarly, emotional abuse was often most commonly perpetrated by patients (Range: 46%–84%) and family/visitors (Range: 39%–69%) across nursing roles and contexts. An exception includes emotional abuse experienced by nurse educators who identified nursing co-workers as the most common source of this type of violence (58%).

## 4. Discussion

We found differences in the prevalence of various types and sources of workplace violence among nurses across a variety of nursing roles and contexts. The most common types of workplace violence reported by BC nurses were emotional abuse, threats of assault and physical assault respectively. Previous research with BC nurses showed similar findings in that emotional abuse (37%), threats of assault (22%) and physical assault (21%) were the most common types of workplace violence respectively [4]. However, our data showed two to ten times higher proportions of all kinds of violence than proportions reported by previous research. For example, while we found 85% of acute care nurses and 84% of direct care nurses reported exposure to emotional abuse in the last year, previous research found 37% of direct care nurses in acute care settings reported this type of workplace violence over the last five shifts [4]. This difference is also notable among less common types of workplace violence such as verbal sexual harassment (this study = 56%–58% vs. previous research = 8%) and sexual assault (this study = 12%–13% vs. previous research = 0.8%) [4]. 

These data may represent actual increases in workplace violence against BC nurses since two decades ago [4]. While different time frames were used to explore workplace violence in these studies, increased trends over time may also represent better detection and reporting. The provincial nurses’ union started a province-wide campaign in 2017 with a view to battle the culture of normalization and raise awareness that violence is NOT part of nurses’ jobs [22]. 

There were differences in the prevalence of workplace violence primarily across nursing roles and sectors. Compared to nurse educators, a higher proportion of direct care nurses and nurse leaders reported exposure to all types of workplace violence. This finding is interpreted in light of the study finding that identified patients and their families/visitors as the most common instigators of workplace violence. Direct care nurses are at the forefront of healthcare delivery providing the majority of patient care with respect to care delivery hours and proximity. Accordingly, these nurses are a primary target of patient and family/visitor frustration when their needs are not met. A recent study found a link between direct care nurses’ heavy workloads and more frequent patient and family complaints escalating into emotional and physical violence towards nurses [5]. Future research should examine the impact of the quality of patient care on workplace violence towards nurses. In addition, more exploration is needed about violence against nurse leaders. There is a dearth of published research on violence against nurses in non-direct care roles. We surmise that a role of nurse leaders may be intervening in situations where patients/families are complaining about their nursing care, placing them at bedsides when emotions, such as anxiety and frustration, are escalating. Research shows staff nurses often manage workplace violence through the support of a colleague and a superior [23]. More specifically, this study found one third of nursing victims discussed incidents with a superior following workplace violence [23]. We suspect, leaders’ involvement in workplace violence management explains their higher report of exposure found in this study. Future research should further explore nurse leaders’ experiences of workplace violence. 

Although increases in all types of violence are concerning, the rise in types of sexual violence from a decade ago is particularly worrisome. A meta-analysis of 41 studies identified an organizational culture of tolerance as one of the most important predictors of sexual harassment. This finding highlights the important role of organizations in preventing workplace violence particularly sexual violence towards nurses [24]. This meta-analysis also found exposure to sexual harassment is linked to poor mental and physical health, life dissatisfaction and PTSD [24].

Compared to community care nurses, a higher proportion of nurses in acute care and long-term care sectors were exposed to workplace violence. This finding can be explained by the shorter patient and family/visitors contact in community care as opposed to the other two sectors. In acute care and long-term care sectors, patients and their families/visitors typically have length of stays long enough to recognize changes in quality of care imposed by work environment conditions such as staffing shortages, heavy workloads and long wait times [5,17,21]. Despite, differences in the conditions of nurses’ works environments across geographical areas [18,19,20], we did not find statistically significant differences in the prevalence of most types of workplace violence across geographical areas. That said, the raw prevalence for most types of workplace violence was slightly higher in rural areas. Accordingly, we suspect that our study did not have sufficient power to detect smaller differences in the prevalence of workplace violence. As discussed by previous workplace violence research, even small violence exposure may have significant, adverse consequences [3,10].

We also found acute care nurses that worked in emergency departments, psychiatric and medical/surgical specialties reported the highest prevalence of almost all types of workplace violence. Earlier research identified these nursing specialties as high-risk areas [7,25,26]. However, there seems to be a shift in the pattern of workplace violence across nursing specialties. In this study, while the highest prevalence of physical assault, emotional abuse and verbal sexual harassment was reported by emergency department nurses, the highest prevalence of sexual assault was reported by medical-surgical nurses. Threats of assault were equally reported by emergency and psychiatry nurses. Previous research with BC nurses found the most common types of workplace violence as: physical assault in medical-surgical specialties; threats of assault, verbal sexual harassment and sexual assault in psychiatry; and emotional abuse in emergency departments [6]. 

One potential explanation for shifting patterns of violence by nursing specialty may be the increase in patient complexities and care needs in under-resourced healthcare environments. One Australian study with hospital nurses found that different combinations of workplace factors (e.g., limited resources, job demands) were predictive of specific types of aggression by patients and by colleagues [27]. For example, increased job demands were associated with patient threats of assault. The researchers proposed that when nurses are pressured by heavy workloads, for example, they have less capacity to respond to patient needs, triggering assault threats [27]. 

As an example, The Canadian Institute of Health Information database showed that in BC between 2014 to 2019, patients experienced above the national average wait times in emergency departments when compared to other provincial emergency department wait times [28]. Thus, the high prevalence of workplace violence against BC nurses in emergency departments may be associated with frustrated patients and families who must wait long hours for assessment and treatment. Additionally, patients living with mental health diagnosis and substance use disorders are one of the most frequent users of emergency services [29]. Research shows the greatest safety and security concerns in emergency departments are dissatisfied patients and behavior health issues [30]. We would like to note, however, that more than half of nurses from ‘low-risk’ areas, such as critical care settings, reported exposure to certain types of workplace violence, suggesting that nurse workplace violence has become a systemic problem across BC acute care settings [31].

Finally, this study found patients and their families were the most common perpetuators of almost all types of workplace violence towards nurses. Although, to our knowledge, this is the first study to explore the prevalence of nurse workplace violence across a variety of nursing roles and contexts, previous national and international research reported similar findings among aggregate samples of nurses [6,7]. We also found a relatively high prevalence of horizontal violence, particularly emotional abuse, among nurses in a variety of roles and contexts. Surprisingly, nursing co-workers were identified as the most prominent source of emotional abuse by nurse educators, more so than patients and their families. While previous research also pointed to the phenomenon of bullying and horizontal violence among nurses [7,32,33,34], we think its high prevalence among educators is attributed to their significant involvement in training and educating their nursing colleagues. 

The findings of this study demonstrate an urgent need for more effective workplace violence prevention initiatives that are role and context specific. In 2019, the House of Commons Standing Committee on Health identified nine recommendations focused on minimizing and eradicating workplace violence among healthcare workers [1]. While some of these recommendations focused on addressing the root causes of workplace violence, others emphasized the importance of existing interventions to better protect the health and safety of healthcare workers and their clients [1]. A recent study of BC mental health and medical-surgical nurses found several of the existing interventions are ineffective [31]. For example, prevention training and personal protective devices, also known as personal alarms, despite their prevalent use, did not positively contribute to nurses’ perceptions of workplace safety. Strategies that enhanced nurses’ perceptions of workplace safety were conversations with leadership and engagement in collaborative problem-solving [31]. Thus, it is essential for leadership to engage staff nurses in policies, decisions and interventions to prevent workplace violence. Researchers, health employers, policy makers and practitioners should work together towards evaluating and implementing interventions that better ensure workplace safety for nurses across a variety of roles and contexts. 

### Limitations

This is the first province-wide study to explore the prevalence of various typologies and sources of workplace violence among nearly 4500 nurses across a variety of roles, sectors, geographical areas and nursing specialties. However, there are several limitations associated with this study. First, the study response rate is low which raises concerns around sampling bias and external validity. Although a high response rate does not ensure representation and vice versa, the findings should be cautiously generalized to other samples and contexts [21]. Second, workplace violence questions asked about exposures over the last year, which raises the possibility of recall bias. Third, responses to workplace violence exposure types were recoded into a binary, ignoring the variance in exposure frequency. Finally, due to the cross-sectional nature of the data, we cannot establish cause and effect between study variables. 

## 5. Conclusions

This study found that the most common types of workplace violence were emotional abuse, threats of assault and physical assault respectively. For most nurses, patients and their families/visitors were the most common instigators of workplace violence. There were differences in the prevalence of various types of workplace violence across nursing roles, healthcare sectors and nursing specialties. Since research with data from two decades ago that examined workplace violence against BC nurses, there have been increases in all types of violence. These shifts may signal actual increases or better detection and reporting. These study findings will help inform workplace interventions that mitigate risk to nurses and other healthcare providers. A premise of this survey study is that nurses are best placed to know what is working for them—or not. Nurses, therefore, should be involved in the design and implementation of workplace interventions suited to their specific contexts and patient populations. 

## Figures and Tables

**Table 1:** Demographic and workplace characteristics (*n* = 4462).

Characteristics	*n*	%
*Age*		
Under 25	190	4.3
25 to 34	1465	33.0
35 to 44	1123	25.3
45 to 54	961	21.7
55 and above	697	15.7
*Gender*		
Female	4071	91.3
Male	379	8.5
Prefer to describe	9	0.2
*Professional Designation* ^1^		
LPN	722	16.2
RN	3467	77.7
RPN	276	5.7
Dually registered (RN/RPN)	17	0.4
*Education*		
Diploma/Certificate	1365	30.6
Undergraduate degree	2132	47.8
Graduate degree	905	20.3
Other	57	1.3
*Overall nursing experience*		
5 years or less	1307	29.4
6 to 10 years	957	21.5
11 to 15 years	777	17.5
16 to 20 years	364	8.2
21 years or more	1044	23.5
*Nursing Role*		
Direct care provider	3995	89.5
Nurse leader	351	7.9
Nurse educator	116	2.6
*Sector*		
Acute care	3277	73.5
Community care	780	17.5
Long-term care	400	9.0
*Geographical Area*		
Urban	2777	62.5
Suburban	781	17.6
Rural	885	19.9
*Acute Care Nursing Specialty (n = 3260)*		
Medical/Surgical	1142	35.0
Critical care	578	17.7
Emergency	468	14.4
Psychiatry	272	8.3
All other units	710	21.8
Multiple units	90	2.8

^1^ LPN, licensed practical nurses; RN, registered nurses; RPN, registered psychiatric nurses.

**Table 2:** Percentage of nurses who reported workplace violence by nursing role, healthcare sector and geographical area.

Key Variables	Physical Assault	Threat of Assault	Emotional Abuse	Verbal Sexual Harassment	Sexual Assault
*Overall sample*	2971 (66.6%)	3497 (78.4%)	3693 (82.8%)	2448 (54.9%)	503 (11.3%)
*Nursing Role*					
Direct care provider	2724 (68.2%)	3180 (79.6%)	3347 (83.8%)	2248 (56.3%)	468 (11.7%)
Nurse leader	214 (61.0%)	267 (76.1%)	277 (78.9%)	171 (48.7%)	35 (10.0%)
Nurse educator	33 (28.4%)	50 (43.1%)	69 (59.5%)	29 (25.0%)	0 (0%)
χ^2^*(24,462)*	85.4 ***	89.8 ***	50.6 ***	50.3 ***	^−1^
*Sector*					
Acute care	2393 (73.0%)	2692 (82.1%)	2795 (85.3%)	1907 (58.2%)	412 (12.6%)
Community care	234 (30.0%)	463 (59.4%)	560 (71.8%)	317 (40.6%)	29 (3.7%)
Long-term care	340 (85.0%)	337 (84.3%)	334 (83.5%)	222 (55.5%)	62 (15.5%)
χ^2^*(24,457)*	591.1 ***	201.9 ***	80.6 ***	78.5 ***	57.1 ***
*Geographical Area*					
Urban	1824 (65.7%)	2148 (77.3%)	2297 (82.7%)	1453 (52.3%)	304 (10.9%)
Suburban	536 (68.6%)	613 (78.5%)	641 (82.1%)	444 (56.9%)	86 (11.0%)
Rural	595 (67.2%)	718 (81.1%)	739 (83.5%)	541 (61.1%)	111 (12.5%)
χ^2^*(24,443)*	2.6	5.7	0.6	22.5 ***	1.8

^1^ No test statistic is provided because a χ^2^ assumption is violated. *** *p* < 0.001.

**Table 3:** Percentage of acute care nurses who reported workplace violence by nursing specialty.

Acute Care Nursing Specialty	Physical Assault	Threat of Assault	Emotional Abuse	Verbal Sexual Harassment	Sexual Assault
Medical/Surgical	987 (86.4%)	1039 (91%)	1021 (89.4%)	808 (70.8%)	228 (20.0%)
Critical care	349 (60.4%)	418 (72.3%)	454 (78.5%)	245 (42.4%)	31 (5.4%)
Emergency	419 (89.5%)	449 (96%)	452 (96.6%)	369 (78.8%)	81 (17.3%)
Psychiatry	210 (77.2%)	261 (96%)	254 (93.4%)	193 (71.0%)	22 (8.1%)
All other units	357 (50.3%)	443 (62.4%)	521 (73.4%)	232 (32.7%)	33 (4.6%)
Multiple specialties	62 (68.9%)	71 (78.9%)	78 (86.7%)	54 (60.0%)	15 (16.7%)
*X^2^ (53,260)*	406.4 ***	386.0 ***	178.4 ***	424.1 ***	140.5 ***

*** *p* < 0.001.

**Table 4:** Percentage of workplace violence that is reported compared by single and multiple sources of workplace violence.

Nursing Role	Physical Assault	Threat of Assault	Emotional Abuse	Verbal Sexual Harassment	Sexual Assault
***Direct care provider***	*n* = 2718	*n* = 3173	*n* = 3339	*n* = 2242	*n* = 465
Patients	72.1%	45.5%	16.9%	65.1%	76.1%
Family/visitors	0.5%	4.3%	3.1%	1.7%	0.4%
Physicians	0.1%	0.3%	0.9%	0.8%	0.6%
Nursing co-workers	0.2%	0.3%	3.3%	1.1%	0.4%
Allied health	0%	0.1%	0.1%	0.3%	0.2%
Management	0%	0.1%	2.0%	0%	0.2%
Other	0.7%	0.6%	1.0%	7.3%	10.1%
Multiple sources	26.5%	48.9%	72.6%	23.8%	11.8%
***Nurse leader***	*n* = 214	*n* = 267	*n* = 277	*n* = 171	*n* = 35
Patients	71.5%	43.8%	11.6%	66.1%	77.1%
Family/visitors	0%	3.7%	3.2%	1.8%	0%
Physicians	0%	0.4%	0.7%	2.3%	0%
Nursing co-workers	0%	0.7%	4.3%	1.8%	2.9%
Allied health	0%	0%	0%	0.6%	0%
Management	0%	0%	3.6%	0%	0%
Other	0.9%	1.1%	1.4%	9.9%	17.1%
Multiple sources	27.6%	50.2%	75.1%	17.5%	2.9%
***Nurse educator***	*n* = 33	*n* = 50	*n* = 69	*n* = 29	*n* = 0
Patients	69.7%	38.0%	7.2%	69.0%	-
Family/visitors	0%	8.0%	1.4%	3.4%	-
Physicians	3.0%	2.0%	2.9%	3.4%	-
Nursing co-workers	0%	4.0%	20.3%	6.9%	-
Allied health	0%	0%	0%	0%	-
Management	0%	2.0%	4.3%	0%	-
Other	0%	4.0%	1.4%	6.9%	-
Multiple sources	27.3%	42.0%	62.3%	10.3%	-
**Sector**					
***Acute care***	*n* = 2389	*n* = 2687	*n* = 2789	*n* = 1904	*n* = 409
Patients	70.0%	41.6%	14.8%	63.8%	77.3%
Family/visitors	0.5%	4.3%	2.8%	1.9%	0.2%
Physicians	0.1%	0.4%	1.1%	1.2%	0.5%
Nursing co-workers	0.1%	0.3%	2.8%	1.0%	0%
Allied health	0%	0%	0%	0.2%	0.2%
Management	0%	0.1%	1.7%	0.1%	0.2%
Other	0.5%	0.3%	0.8%	7.7%	10.0%
Multiple sources	28.8%	53.0%	75.9%	24.1%	11.5%
***Community care***	*n* = 233	*n* = 462	*n* = 559	*n* = 315	*n* = 29
Patients	76.0%	53.9%	18.4%	67.6%	65.5%
Family/visitors	0.4%	6.1%	2.9%	1.3%	3.4%
Physicians	0%	0%	0.5%	0.3%	0%
Nursing co-workers	0.4%	0.4%	7.9%	0.6%	0%
Allied health	0%	0.6%	0.7%	1.0%	0%
Management	0%	0.2%	4.3%	0%	0%
Other	0.9%	2.2%	2.5%	5.7%	13.8%
Multiple sources	22.3%	36.6%	62.8%	23.5%	17.2%
***Long-term care***	*n* = 339	*n* = 336	*n* = 333	*n* = 221	*n* = 62
Patients	83.8%	62.8%	25.5%	73.8%	74.2%
Family/visitors	0%	2.1%	5.7%	.5%	0%
Physicians	0.3%	0%	.3%	0%	1.6%
Nursing co-workers	0.3%	1.2%	3.9%	3.6%	4.8%
Allied health	0%	0%	0%	0%	0%
Management	0%	0%	2.7%	0%	0%
Other	2.1%	.9%	1.5%	7.7%	12.9%
Multiple sources	13.6%	33.0%	60.4%	14.5%	6.5%
**Geographical area**					
***Urban***	*n* = 1822	*n* = 2145	*n* = 2293	*n* = 1451	*n* = 302
Patients	71.1%	45.0%	17.6%	65.1%	77.5%
Family/visitors	0.7%	4.8%	3.2%	2.1%	0.3%
Physicians	0.1%	0.3%	0.8%	0.9%	0%
Nursing co-workers	0.2%	0.4%	3.9%	1.3%	0.3%
Allied health	0%	0.1%	0.2%	0.3%	0%
Management	0%	0.2%	2.0%	0.1%	0.3%
Other	0.7%	0.7%	1.0%	7.7%	11.6%
Multiple sources	27.3%	48.4%	71.3%	22.5%	9.9%
***Suburban***	*n* = 534	*n* = 611	*n* = 639	*n* = 442	*n* = 85
Patients	69.7%	42.4%	13.6%	64.3%	72.9%
Family/visitors	0.4%	3.6%	3.0%	0.9%	1.2%
Physicians	0.2%	0%	0.3%	1.1%	0%
Nursing co-workers	0%	0.3%	3.1%	0.9%	2.4%
Allied health	0%	0%	0%	0.2%	1.2%
Management	0%	0.2%	1.7%	0%	0%
Other	0.4%	0.3%	1.1%	8.1%	9.4%
Multiple sources	29.4%	53.2%	77.2%	24.4%	12.9%
***Rural***	*n* = 593	*n* = 716	*n* = 737	*n* = 539	*n* = 111
Patients	77.1%	48.0%	14.8%	66.0%	74.8%
Family/visitors	0%	3.5%	2.8%	1.1%	0%
Physicians	0.2%	0.6%	1.9%	0.9%	2.7%
Nursing co-workers	0.2%	0.3%	3.5%	1.1%	0%
Allied health	0%	0.1%	0.1%	0.4%	0%
Management	0%	0%	3.1%	0%	0%
Other	1.0%	0.6%	1.2%	6.3%	9.0%
Multiple sources	21.6%	46.9%	72.5%	24.1%	13.5%

**Table 5:** Percentage of workplace violence compared individually by source.

Nursing Role	Physical Assault	Threat of Assault	Emotional Abuse	Verbal Sexual Harassment	Sexual Assault
***Direct care provider***	*n* = 2718	*n* = 3173	*n* = 3339	*n* = 2242	*n* = 465
Patients	98.5%	94.2%	82.8%	88.4%	88.0%
Family/Visitors	25.9%	52.3%	64.1%	22.3%	10.8%
Physicians	2.0%	1.7%	23.7%	3.5%	1.1%
Nursing co-workers	2.7%	2.4%	31.4%	3.9%	1.1%
Allied health	0.6%	0.8%	4.9%	1.1%	0.6%
Management	1.4%	1.3%	23.1%	0.8%	0.4%
Other	0.9%	1.0%	1.9%	7.9%	11.0%
***Nurse leader***	*n* = 214	*n* = 267	*n* = 277	*n* = 171	*n* = 35
Patients	*98.1%*	*93.3%*	*76.2%*	*83.6%*	*80.0%*
Family/Visitors	24.3%	52.1%	63.5%	15.8%	2.9%
Physicians	1.4%	3.0%	26.0%	5.3%	0%
Nursing co-workers	3.7%	6.4%	41.9%	5.3%	2.9%
Allied health	0.5%	1.9%	8.7%	1.2%	0%
Management	2.8%	4.1%	30.0%	0.6%	0%
Other	0.9%	1.5%	2.5%	9.9%	17.1%
***Nurse educator***	*n* = 33	*n* = 50	*n* = 69	*n* = 29	*n* = 0
Patients	93.9%	78.0%	46.4%	75.9%	0%
Family/visitors	24.2%	48.0%	39.1%	10.3%	0%
Physicians	12.1%	6.0%	36.2%	6.9%	0%
Nursing co-workers	9.1%	8.0%	58.0%	10.3%	0%
Allied health	0%	0%	8.7%	0%	0%
Management	3.0%	2.0%	26.1%	0%	0%
Other	0%	6.0%	4.3%	6.9%	0%
**Sector**					
***Acute care***	*n* = 2389	*n* = 2687	*n* = 2789	*n* = 1904	*n* = 409
Patients	98.7%	94.3%	83.8%	87.5%	88.8%
Family/Visitors	28.0%	56.7%	67.5%	23.1%	10.5%
Physicians	2.1%	1.7%	27.8%	4.0%	1.0%
Nursing co-workers	2.7%	1.9%	31.4%	3.5%	0.2%
Allied health	0.5%	0.6%	4.6%	0.9%	0.5%
Management	1.3%	1.3%	22.3%	0.7%	0.5%
Other	0.6%	0.6%	1.5%	8.0%	10.5%
***Community care***	*n* = 233	*n* = 462	*n* = 559	*n* = 315	*n* = 29
Patients	97.9%	90.5%	71.7%	90.5%	82.8%
Family/Visitors	21.0%	40.3%	48.5%	20.6%	17.2%
Physicians	2.6%	2.8%	14.5%	2.9%	0%
Nursing co-workers	3.4%	5.2%	37.7%	3.2%	3.4%
Allied health	1.3%	2.4%	7.2%	2.2%	3.4%
Management	2.1%	2.8%	30.4%	1.3%	0%
Other	1.7%	3.7%	3.9%	8.6%	17.2%
***Long-term care***	*n* = 339	*n* = 336	*n* = 333	*n* = 221	*n* = 62
Patients	97.1%	95.2%	80.5%	88.2%	80.6%
Family/Visitors	12.7%	33.0%	56.2%	11.3%	4.8%
Physicians	1.5%	1.2%	10.2%	1.8%	1.6%
Nursing co-workers	3.8%	6.2%	35.1%	10.0%	6.5%
Allied health	1.2%	1.2%	8.1%	0.9%	0%
Management	2.4%	1.8%	22.8%	1.4%	0%
Other	2.1%	1.5%	3.3%	7.7%	14.5%
**Geographical area**					
***Urban***	*n* = 1822	*n* = 2145	*n* = 2293	*n* = 1451	*n* = 302
Patients	98.3%	93.1%	81.3%	86.9%	87.4%
Family/Visitors	26.7%	52.3%	62.7%	21.0%	9.6%
Physicians	2.0%	2.1%	23.9%	4.0%	0.3%
Nursing co-workers	3.0%	2.8%	32.8%	5.0%	1.0%
Allied health	0.6%	0.8%	5.9%	1.3%	0.3%
Management	1.4%	1.6%	22.8%	0.9%	0.3%
Other	0.9%	1.2%	2.0%	8.4%	12.3%
***Suburban***	*n* = 534	*n* = 611	*n* = 639	*n* = 442	*n* = 85
Patients	98.7%	95.6%	83.9%	88.7%	85.9%
Family/Visitors	27.7%	56.0%	68.9%	23.5%	11.8%
Physicians	3.0%	0.7%	25.4%	2.9%	0%
Nursing co-workers	3.4%	2.5%	31.6%	2.5%	2.4%
Allied health	0.6%	0.8%	3.8%	1.1%	2.4%
Management	2.6%	1.5%	24.3%	0.5%	0%
Other	0.4%	0.5%	2.3%	8.6%	10.6%
***Rural***	*n* = 593	*n* = 716	*n* = 737	*n* = 539	*n* = 111
Patients	98.7%	94.8%	80.6%	90.2%	88.3%
Family/Visitors	20.9%	49.4%	61.6%	22.1%	10.8%
Physicians	1.3%	2.1%	24.2%	3.5%	3.6%
Nursing co-workers	2.2%	2.9%	33.5%	3.0%	0.9%
Allied health	0.7%	1.1%	4.5%	0.6%	0%
Management	0.8%	1.4%	25.6%	0.9%	0.9%
Other	1.2%	1.1%	1.9%	6.7%	9.9%
